# Camouflage Effects of Various Colour-Marking Morphs against Different Microhabitat Backgrounds in a Polymorphic Pygmy Grasshopper *Tetrix japonica*

**DOI:** 10.1371/journal.pone.0011446

**Published:** 2010-07-06

**Authors:** Kaori Tsurui, Atsushi Honma, Takayoshi Nishida

**Affiliations:** Laboratory of Insect Ecology, Graduate School of Agriculture, Kyoto University, Sakyo, Kyoto, Japan; University of Bristol, United Kingdom

## Abstract

**Background:**

Colour-marking polymorphism is widely distributed among cryptic species. To account for the adaptive significance of such polymorphisms, several hypotheses have been proposed to date. Although these hypotheses argue over the degree of camouflage effects of marking morphs (and the interactions between morphs and their microhabitat backgrounds), as far as we know, most empirical evidence has been provided under unnatural conditions (i.e., using artificial prey).

**Methodology/Principal Findings:**

*Tetrix japonica*, a pygmy grasshopper, is highly polymorphic in colour-markings and occurs in both sand and grass microhabitats. Even within a microhabitat, *T. japonica* is highly polymorphic. Using humans as dummy predators and printed photographs in which various morphs of grasshoppers were placed against different backgrounds, we addressed three questions to test the neutral, background heterogeneity, and differential crypsis hypotheses in four marking-type morphs: 1) do the morphs differ in the degree of crypsis in each microhabitat, 2) are different morphs most cryptic in specific backgrounds of the microhabitats, and 3) does the morph frequency reflect the degree of crypsis?

**Conclusions/Significance:**

The degree of camouflage differed among the four morphs; therefore, the neutral hypothesis was rejected. Furthermore, the order of camouflage advantage among morphs differed depending on the two types of backgrounds (sand and grass), although the grass background consistently provided greater camouflage effects. Thus, based on our results, we could not reject the background heterogeneity hypothesis. Under field conditions, the more cryptic morphs comprised a minority of the population. Overall, our results demonstrate that the different morphs were not equivalent in the degree of crypsis, but the degree of camouflage of the morphs was not consistent with the morph frequency. These findings suggest that trade-offs exist between the camouflage benefit of body colouration and other fitness components, providing a better understanding of the adaptive significance of colour-markings and presumably supporting the differential crypsis hypothesis.

## Introduction

Colour-marking polymorphism is widely distributed among cryptic species. [1] Moreover, some species are highly polymorphic in colour and markings even within a single population [2]. Camouflage is one of the most common forms of defensive colouration [3] against visually hunting predators. [4], [5], [6], [7] Many animal species have contrasting body markings; despite this, they often appear to be rather cryptic against their natural backgrounds, at least to the human eye. To account for the adaptive significance of such polymorphisms, at least four hypotheses have been proposed to date: the neutral hypothesis [8], the background heterogeneity hypothesis [9], [10], the search image hypothesis [1], [11], and the differential crypsis hypothesis (inferred by Forsman 1998). [12] The neutral hypothesis posits that each colour-marking polymorphism provides the same cryptic effects, and thus all morphs are neutral in terms of fitness. The background heterogeneity hypothesis states that each morph has the advantage within a specific background environment, and thus species occurring in highly heterogeneous environments exhibit polymorphisms. In contrast, the differential crypsis hypothesis assumes a differential degree of crypsis among polymorphic morphs; therefore, trade-offs between the degree of crypsis and other fitness components, such as mating advantage or thermoregulation ability [13], are necessary to maintain the polymorphism. Although colour-marking polymorphisms are not rare, empirical evidence remains limited for which hypothesis is applicable to real organisms in the wild.

Pygmy grasshoppers are typical examples of such polymorphic species. [14], [15] *Tetrix japonica* occurs in both grass and sand microhabitats and exhibits large variation in body colouration and markings. [15] Preliminary observations have suggested that the proportion of morphs differs between the two microhabitats, and even within a single microhabitat, several types of morphs can co-occur. Consequently, *T. japonica* is a suitable organism to examine the above hypotheses regarding colour-marking polymorphisms.

To examine whether the markings affect camouflage and whether the camouflage effect depends on background type (sand and grass microhabitat), we conducted detection task experiments on grasshoppers against different natural backgrounds using humans as dummy predators. In such experiments, humans offer several advantages over real predators such as wild birds. The behaviour of wild animals can be strongly influenced by their degree of hunger, previous experience, and the experimental environment, whereas humans are far less affected by these factors. [16] Moreover, a recent study using humans as dummy predators yielded results that were virtually identical to the findings of earlier studies using bird predators. [17], [18] In addition, we conducted a field census of the polymorphism in neighbouring microhabitats (sand and grass) to confirm whether the degree of camouflage reflects the grasshopper morph frequency.

## Materials and Methods

### *Tetrix japonica*

Pygmy grasshoppers (Tetriginae) are characterised by both a long pronotum that extends beyond the apex of the abdomen and highly reduced forewings. [19] *Tetrix japonica* is a small grasshopper (males, 7.7–9.5 mm; females, 9.0–13.0 mm) that usually inhabits relatively dry places (soil moisture  = 30–40%; Atsushi Honma unpublished data) compared to other sympatric Tetriginae species. *Tetrix japonica* exhibits extraordinary variation in the colour and markings of the pronotum. [15] Even within a single population, the basal body colouration varies from blackish brown to yellowish brown to pale grey. Some grasshoppers are bi-coloured, with whitish and blackish markings on the dorsal surface of the pronotum. In contrast, some *T. japonica* have no markings, whereas others have spots or other distinct patterns on the pronotum.

### Study Site

The study sites were two adjacent terraced fallow fields (“grass” and “sand” microhabitats) located about 30 m apart in Iwakura, a northern suburb of Kyoto, Japan (135°47.6′W, 35°5.7′N). The fields had been fallow for at least 9 years. The grass microhabitat (approximately 136 m^2^ in area) consisted of marsh and thickets that were clear-cut once a year in the autumn and were dominated by Japanese millet, *Echinochola crus-galli* var., and annual bluegrass, *Poa annua*. The sand microhabitat (approximately 62 m^2^ in area) primarily consisted of bare pebbles next to small, short thickets dominated by *P. annua*. We observed many visually hunting predator species landing on and foraging in the fallow fields; these included Japanese pied wagtails *Motacilla grandis*, Siberian meadow buntings *Emberiza cioides*, grey starlings *Sturnus cineraceus*, and dusky thrushes *Turdus naumanni* (from autumn to spring). Frogs and spiders were also abundant.

### Definition of Colour-Marking Morphs

**Figure 1:**
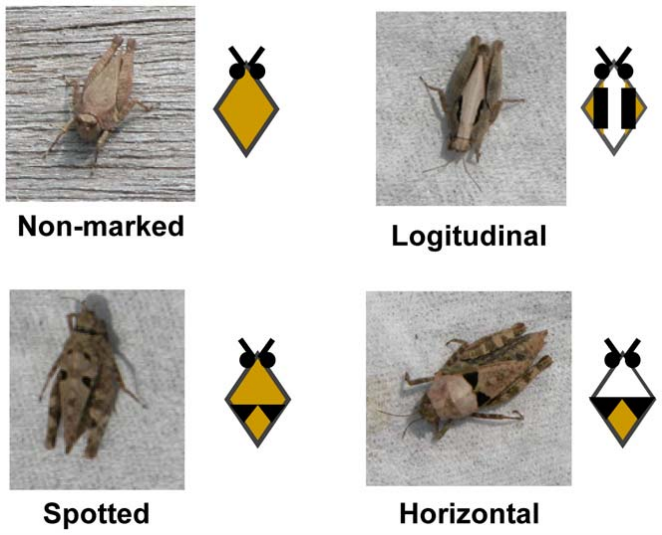
Morphs of pygmy grasshoppers (*Tetrix japonica*) classified by type of markings. Pygmy grasshoppers (*Tetrix japonica*) used in the experiments.

We categorised *T. japonica* morphs into four groups based on type of markings: non-marked morphs with no markings at all on the body, spotted morphs with round markings at the lower-middle part of the pronotum, longitudinal morphs (whitish along the longitudinal axis of the pronotum and grey-brown at the other part of the pronotum), and horizontal morphs (whitish at the forepart and grey-brown at the rear half) (Figure 1). The number of spots varied among spotted morphs; therefore, we used only two-spotted individuals as ‘spotted morphs’ in the detection task experiment. In the field survey of the frequency of grasshopper morphs, spotted morphs included all grasshoppers with any number of spots on mono-coloured basal colouration. Longitudinal morphs resembled withered grass, and horizontal morphs appeared quite similar to the white and black pebbles of the sand microhabitat.

### Detection Task Experiment

For the detection task experiments, the detection time of grasshopper images by the human predators was used as a proxy for the survival probability of grasshoppers. Adult *T. japonica* grasshoppers were captured using random sweeps of an insect net within the sand and grass microhabitats. Each grasshopper was anaesthetised with CO_2_ and then photographed against two different backgrounds. The two backgrounds consisted of the ground at the sand and grass microhabitats in which the grasshoppers were captured. Two frames (A4 size: 210×297 mm) were set out in each microhabitat and were divided into 48 cells (6×8). Within a randomly chosen cell, one grasshopper was placed in a natural position. Each grasshopper was photographed within the same frame from a vertical height of 0.25 m. Photographs (2560×1920 pixels; Nikon Coolpix 5700) were taken under natural light conditions on a sunny day without flash, saved as uncompressed TIFF files, and printed on A4-sized (210×297 mm) PPC paper on an Epson LP-9000C (600 dpi) colour laser printer. The printed grasshopper images were approximately the same size as the real grasshoppers.

We measured the time (up to 1 min) per photograph taken by each of 18 humans to detect a grasshopper in each of the 39 photographs (15 non-marked morphs, 14 spotted morphs, 7 longitudinal morphs, and 3 horizontal morphs; Table S1). Each participant was presented with all photographs. The morph frequencies in the experiment were chosen to be approximately equal to those in the field on the day and time of the photo shoot. Preliminary experiments have suggested that the detection time of the grasshoppers against the grass microhabitat is longer than that against the sand microhabitat and that grass microhabitat trials require more concentration (on the part of the human dummy predators) than do the sand trials (Tsurui, unpublished data). Thus, to maintain the motivation of the human predators, the number of grass trials was half that of the sand trials (26 sand and 13 grass backgrounds per human; Table S1). The human predators were undergraduate students in the Faculty of Agriculture, Kyoto University, and they were not informed of the experimental goals. No subjects were allowed to participate more than once.

### UV Reflectance of *T. japonica*

Humans and insectivorous birds such as great tits possess very similar visual abilities for prey detection. [17], [18] The major difference in vision between birds and humans is that humans cannot recognise ultraviolet (UV), whereas birds can. To better characterise this potential difference in the perception of grasshoppers, we determined the UV reflectance of *T. japonica*. The UV and visible light spectrum of the pronotum of living grasshoppers were measured using a UV-visible recording spectrophotometer (Ocean Optics USB4E01445). Based on the measurements of the UV and visible light spectrum of *T. japonica*, the UV reflectance of the grasshoppers was relatively low (less than 5%). These results suggest that the range of UV reflectance did not affect the results of the detection task experiments. Therefore, humans were adequate representatives of *T. japonica* natural predators (Figure S1).

### Problems Associated with the Use of Printed Photographs

The perceived grey value and colour contrasts of prey in the printed photographs may differ from those recorded digitally, because the printing process can produce other non-linear transformation effects of colour as well as luminance. [20] Thus, the perceived luminance and colour contrasts of prey in the printed photographs may differ from those on the computer slides or from those observed directly. This problem cannot be overcome even if cameras are precisely calibrated. To minimise such effects, Fraser et al. (2007) [17] and Webster et al. (2009) [18] conducted a series of experiments using computer slides instead of printed materials, and both research groups obtained very similar results using the two methods. We recognise that our experimental procedures involved similar problems; however, based on the results of Fraser et al. (2007) [17] and Webster et al. (2009) [18], such effects were likely to be minor.

### Field Survey of the Frequency of Grasshopper Morphs

Grasshoppers were collected using random sweeps of an insect net in fallow fields. To avoid putative effects of dispersal between different microhabitats, no samplings were conducted at the border of each microhabitat. Grasshopper data were collected on 19 June 2005, around noon, when insects were active after sun basking. For the present study, only adults were used.

### Data Analysis

Survival analysis was performed using Cox proportional hazards regression [21], [22], [23], a semi-parametric form of survival analysis that assumes all treatments have the same-shaped hazard functions but makes no specific assumptions about the nature of the distribution. This method is ideally suited for censored data and the non-uniform changes in predation risk with respect to time of day that are evident in such data. [24], [25] Significance was tested using the likelihood ratio test, and pairwise contrasts with sequential Bonferroni correction [26], [27] was used to compare specific marking morphs. In the first analysis, the model included type of marking, background, and their interaction as fixed effects and human predator ID as a random effect. In the second analysis, the model for pairwise comparisons of the specific morphs for each background included type of marking as a fixed effect and human predator ID as a random effect. Both analyses were conducted using the R statistical environment, version 2.10.1 (http://cran.r-project.org/). [28]

The difference in grasshopper frequency of each morph between the two microhabitats was tested using Pearson's Chi-squared test with Yate's correction (R, ver. 2.10.1). [28]

## Results

### The Degree of Camouflage Conferred by Marking Type in Different Backgrounds

**Figure 2:**
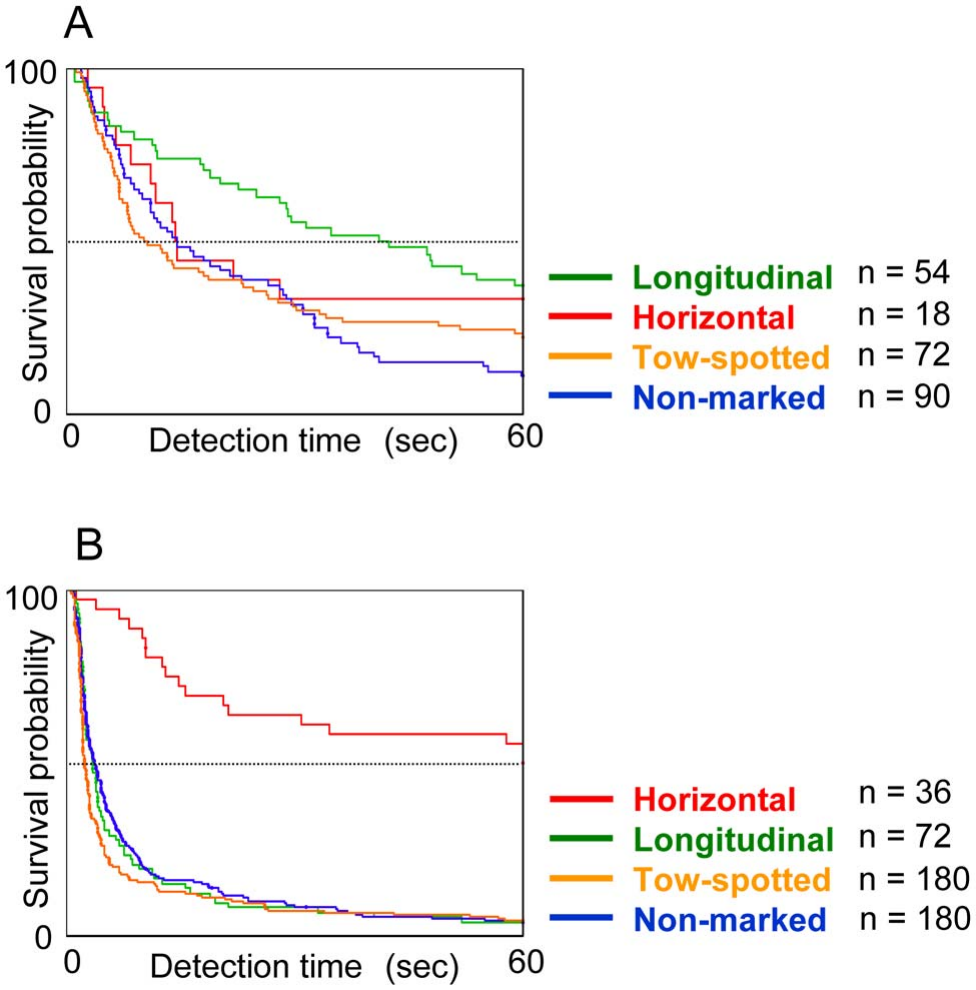
Survival curves of marking-type morphs against grass and sand backgrounds. Curves are the probabilities of surviving human detection as a function of time, based on Kaplan–Meier estimates to account for survival to the end of the experimental period (60 s). (A) Survival curves against grass backgrounds. (B) Survival curves against sand backgrounds. Non-marked morphs, blue lines; spotted morphs, orange lines; longitudinal morphs, green lines; horizontal morphs, red lines.

The survival model was significant (likelihood χ^2^ = 214, *df* = 8.96 *p*<0.001). Detection time was significantly affected by marking type, background, and their interaction (marking type: likelihood χ^2^ = 91.633, *df* = 3, *p*<0.001; background: likelihood χ^2^ = 124.26, *df* = 1, *p*<0.001; interaction: likelihood χ^2^ = 21.426, *df* = 3, *p*<0.001). Human predator effects were also significant (human predator as a random effect: χ^2^ = 4.6918, *df* = 0, *p*<0.001), but this effect was not relevant to our hypotheses. In addition, the order of crypsis depended in part on the background (Table 1). With the exception of horizontal morphs, grasshoppers were significantly less detected against the grass background than against the sand background (Table 2). Within the grass microhabitat, longitudinal morphs were the least detected (Table 1-a, Figure 2-a). Against the sand background, however, the order of crypsis was reversed. Horizontal morphs were the least detected (Table 1-b, Figure 2-b) against the sand microhabitat. Consequently, non-marked and spotted morphs were more conspicuous among morphs against both grass and sand backgrounds (Table 1-a, b). In contrast, horizontal morphs realised a strong camouflage effect against both the sand and grass backgrounds, although they tended to be less detected against the grass background than against the sand background (Table 2). These results indicate that some morphs of *T. japonica* significantly differ in their level of crypsis. Furthermore, compared to the sand background, the grass background provided a stronger camouflage effect for grasshoppers regardless of the morph type.

**Table 1:** Pairwise contrasts among morphs.

(a) Grass background						
	Non-marked	Spotted	Horizontal	Longitudinal	median (seconds)	Likelihood ratio test with Bonferroni correction
Non-marked		0.81	0.18	0.00032	14.6	a
Spotted			0.24	0.0026	10.47	a
Horizontal				0.38	14.4	a
Longitudinal					41.1	b

(b) Sand background						
	Non-marked	Spotted	Horizontal	Longitudinal	median (seconds)	Likelihood ratio test with Bonferroni correction
Non-marked		0.026	<0.0001	0.77	3.6	a
Spotted			<0.0001	0.17	2.34	a
Horizontal				<0.0001	60	b
Longitudinal					3.42	a

**Table 2:** The effect of background on crypsis for each morph.

	Morph	Better camouflaged background	χ^2^	df	p-value
Marking type					
	Non-marked	grass	33.9	1	<0.0001
	Spotted	grass	51.7	1	<0.0001
	Horizontal	tended toward grass	2.28	1	0.13
	Longitudinal	grass	43.9	1	<0.0001

### Frequency of Morphs Conferred by Marking Type in the Grass and Sand Microhabitats

**Figure 3:**
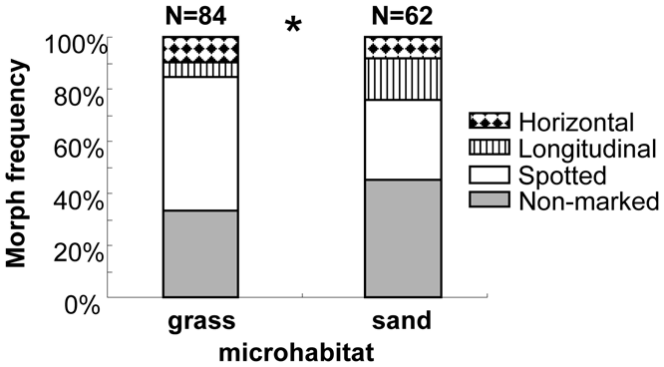
Morph frequency at the study sites (grass and sand microhabitats).

The frequency of *T. japonica* morphs with various types of markings differed significantly between the grass and sand microhabitats (Pearson's Chi-squared test; χ^2^ = 8.53, *df* = 3, *p* = 0.0363; Figure 3). In both microhabitats, the spotted morphs and non-marked morphs were dominant (grass-spotted, 51.2%; grass-non-marked, 33.3%; sand-spotted, 30.6%; sand-non-marked, 45.2%), whereas the longitudinal morphs and horizontal morphs were rare (grass-horizontal, 9.5%; grass- longitudinal, 6.0%; sand-horizontal, 8.1%; sand-longitudinal, 16.1%) These results indicate that the more cryptic morphs are not more common in either the grass or sand microhabitat. Furthermore, longitudinal morphs tended to be more common in the sand microhabitat where they were more conspicuous, although the pattern was not significant (Pearson's Chi-squared test with Yate's continuity correction; χ^2^ = 2.98, *df* = 1, *p* = 0.084; Figure 3). In contrast, spotted morphs were significantly more common in the grass microhabitat where they were more cryptic (Pearson's Chi-squared test with Yate's continuity correction; χ^2^ = 5.3506, *df* = 1, *p* = 0.021; Figure 3). The frequencies of horizontal morphs and spotted morphs did not differ between the grass and sand microhabitats (Pearson's Chi-squared test with Yate's continuity correction; non-marked: χ^2^ = 1.64, *df* = 1, *p* = 0.203; horizontal: χ^2^ = 0.0183, *df* = 1, *p* = 0.9904; Figure 3).

## Discussion

To account for the adaptive significance of colour-marking polymorphisms in terms of camouflage, we considered three hypotheses: the neutral hypothesis [8], the background heterogeneity hypothesis [9], [10], and the differential crypsis hypothesis (inferred by Forsman 1998) [12]. Our experiments indicated that each colour-marking morph differed in terms of the degree of crypsis. Thus, the neutral hypothesis could not explain the adaptive significance of colour-marking polymorphism in *T. japonica.* However, the order of camouflage advantage among morphs differed depending on the two types of backgrounds (sand and grass), although the grass background consistently provided greater camouflage effects. Thus, based on our results, we could not reject the background heterogeneity hypothesis. On the other hand, our field survey revealed that the more cryptic morphs were rarer in each microhabitat. The background heterogeneity hypothesis cannot explain this pattern in morph frequency. Furthermore, each morph was not always common where they were more cryptic. These findings suggest that trade-offs exist between the camouflage benefit of body colouration and other fitness components, providing a better understanding of the adaptive significance of colour-markings and presumably supporting the differential crypsis hypothesis.

The most puzzling result in our study was that each morph occurred not only in the microhabitat where it was most cryptic, but also in the microhabitat where it was easily detectable. For example, longitudinal morphs occurred in the grass microhabitat where they enjoy a strong effect of camouflage as well as in the sand microhabitat where they are conspicuous. In contrast, spotted morphs were common in the grass microhabitat where they were more cryptic. These patterns may be attributable to the reproductive behaviour of pygmy grasshoppers, in that mating generally occurs on bare ground, such as that found in sand microhabitats. [29] If this is the case, trade-offs may exist between mating and camouflage. Further studies are necessary to determine the relationship between marking morphs and sex-related mating behaviour. Another potential explanation is gene flow or dispersal, which may counteract locally varying selection favouring the most cryptic morph, thereby contributing to the maintenance of colour-marking polymorphisms within populations of *T. japonica*. A similar pattern has been observed in a polymorphic isopod. [10] However, in *T. japonica*, longitudinal and horizontal morphs were relatively rare at the study sites, despite their camouflage advantage (Figure 2). Moreover, gene flow or dispersal cannot explain the presence of less camouflaged morphs. Considering these results, one logical hypothesis is that the contrasting markings incur high fitness costs. In fact, colour patterns in a congeneric species, *Tetrix undulata*, were correlated with many factors potentially related to fitness, such as body size [30], reproductive schedule [2], and thermoregulatory behaviour [31]. To more comprehensively understand the adaptive significance of colour-marked morphs in nature, experiments are ongoing to detect the fitness costs of contrasting markings in relation to sexual selection and thermoregulation. According to our observations, horizontal morphs or longitudinal morphs were often courted and mounted by conspecific males. If this is the case, they may suffer from the energy costs and risk of injury from such sexual harassment. [32] The thermoregulation hypothesis would be supported by a clear latitudinal cline in males, with a greater proportion of non-marked morphs in southern areas of Japan. [33] If contrasting markings accelerate the speed of body heating, they may affect fitness costs through thermoregulation. For these reasons, we suggest that these fitness costs would drive cryptic morphs, such as the horizontal morphs and longitudinal morphs, to be rare in the population. The morph frequencies in both microhabitats were maintained, and that the horizontal morphs and longitudinal morphs were rare, at least from 2005 to 2009 (Tsurui, unpublished data). Thus, negative frequency-dependent selective pressures such as apostatic predation [1] and non-random mating [34] may contribute to the maintenance of the morph frequency of *T. japonica*. Further study of the maintenance of rare morphs is clearly warranted.

### Camouflage Effect of Contrasting Markings

Concerning the colour of markings, longitudinal morphs and horizontal morphs had contrasting white markings, whereas non-marked morphs and spotted morphs did not (Figure 1). Morphs with contrasting white markings (longitudinal and horizontal morphs) were more cryptic than those without them (non-marked and spotted morphs). However, the longitudinal and horizontal morphs differed in the degree of crypsis against different backgrounds: longitudinal morphs exhibited a higher degree of crypsis against a grass background than did horizontal morphs (Figure 2-a), and horizontal morphs had a much greater degree of crypsis against a sand background than did longitudinal morphs (Figure 2-b).

These results can be intuitively explained by interactive cryptic effects between the shapes of the contrasting markings and attributes of the background. The thin, whitish-yellow, longitudinal markings of longitudinal morphs appear quite similar to withered grass; thus, they can enhance the cryptic effect against grass backgrounds. In contrast, horizontal morphs strongly resembled combinations of pebbles and their shadows, which were abundant components of sand backgrounds. Similar tactics to enhance crypsis may also be prevalent in other species within this system. For example, among inhabitants of grass habitats, whitish longitudinal markings often occur on the back or sides of bodies in many species of grasshoppers, spiders, other arthropods, and even snakes. Whitish longitudinal markings may be generally effective to avoid detection by visual predators in grasslands. Contrasting colourations, similar to those of horizontal morphs, may be common among inhabitants of bare ground dominated by pebbles and coarse-grained soil. [5] For example, chicks of the ringed plover, which inhabit the bare ground of dry riverbeds or the seashore, have a series of strongly contrasting black and white markings on the head, throat, and neck. [5] Such contrasting markings work as disruptive colouration when the markings reach the edge of an organism's body. [35] Similarly, both the contrasting white areas (horizontal or longitudinal lines) of the basal colourations and black spots of *T. japonica* always reach the body outline (see Figure 1), and bi-coloured morphs were always less detectable. Thus, the camouflage effect of *T. japonica* may be attained through a disruptive effect. More experiments are currently underway to confirm the disruptive effect of the contrasting basal colourations and markings of *T. japonica*.

The present study also revealed that the camouflage effects of the backgrounds themselves differ greatly, with strong camouflage effects of the grass background and weaker effects of the sand background. Consequently, potentially poorly camouflaged morphs can attain higher levels of camouflage against the grass background, even without precisely matching it. In our experiments, the grass background was highly heterogeneous compared to the sand background. Bond and Kamil (2006) [36] examined relationships between spatial heterogeneity and prey recognition by predators, and they concluded that colour morphs in less heterogeneous backgrounds were more readily detected than those in more heterogeneous backgrounds, even at the same level of background matching. Both our findings and those of Bond and Kamil (2006) [36] highlight the importance of the interactive effects of prey colouration and backgrounds, particularly in the degree of heterogeneity, on predator cognition for understanding camouflage due to colour-markings in the field. Thus, attributes of the background, such as colour, patch size, or shape, and the number of colour components, may affect the background dependence of the camouflage effect mediated by colour-markings.

In studies such as ours, the difference in colour vision between humans and natural predators is problematic. We demonstrated that *T. japonica* has relatively low UV reflectance; however, the presence/absence of UV detection is only one component of the differences between the vision of humans and other predators. Colour discrimination throughout the spectrum (including the human visible spectrum) differs between birds and humans, and probably even among bird species [37], as does acuity and contrast sensitivity. Consequently, in a strict sense, the present study could not fully reveal the influence of colouration or contrast of markings on camouflage. In this context, future studies must consider the colour sense of true predator species.

## Supporting Information

Table S1 The number of photographs used in the detection task experiment for each human predator.
(0.03 MB DOC)

Figure S1 UV ad visible reflectance of *T. japonica*. Arrows show the body parts where reflectance spectra were taken.
(1.62 MB PPT)
