# Supplementary material for: Camouflage Effects of Various Colour-Marking Morphs against Different Microhabitat Backgrounds in a Polymorphic Pygmy Grasshopper Tetrix japonica
Source: PLoS One. 2010 Jul 6;5(7):e11446. doi: 10.1371/journal.pone.0011446 (PMC2897885; doi:10.1371/journal.pone.0011446)
Supplement: Table S1 — The number of photographs used in the detection task experiment for each human predator. (0.03 MB DOC) [file pone.0011446.s001.doc]

**Table S1**

| **Background** |  | **Morphs** | |  |  |
| --- | --- | --- | --- | --- | --- |
|  | **Non-marked** | **Spotted** | **Horizontal** | **Longitudinal** | **Total** |
| **Sand** | 11 | 9 | 4 | 2 | 26 |
| **Grass** | 4 | 5 | 3 | 1 | 13 |
| **Total** | 15 | 14 | 7 | 3 | **39** |
