# Supplementary material for: Camouflage Effects of Various Colour-Marking Morphs against Different Microhabitat Backgrounds in a Polymorphic Pygmy Grasshopper Tetrix japonica
Source: PLoS One. 2010 Jul 6;5(7):e11446. doi: 10.1371/journal.pone.0011446 (PMC2897885; doi:10.1371/journal.pone.0011446)
Supplement: Figure S1 — UV ad visible reflectance of T. japonica. Arrows show the body parts where reflectance spectra were taken. (1.62 MB PPT) [file pone.0011446.s002.ppt]

## Slide 1
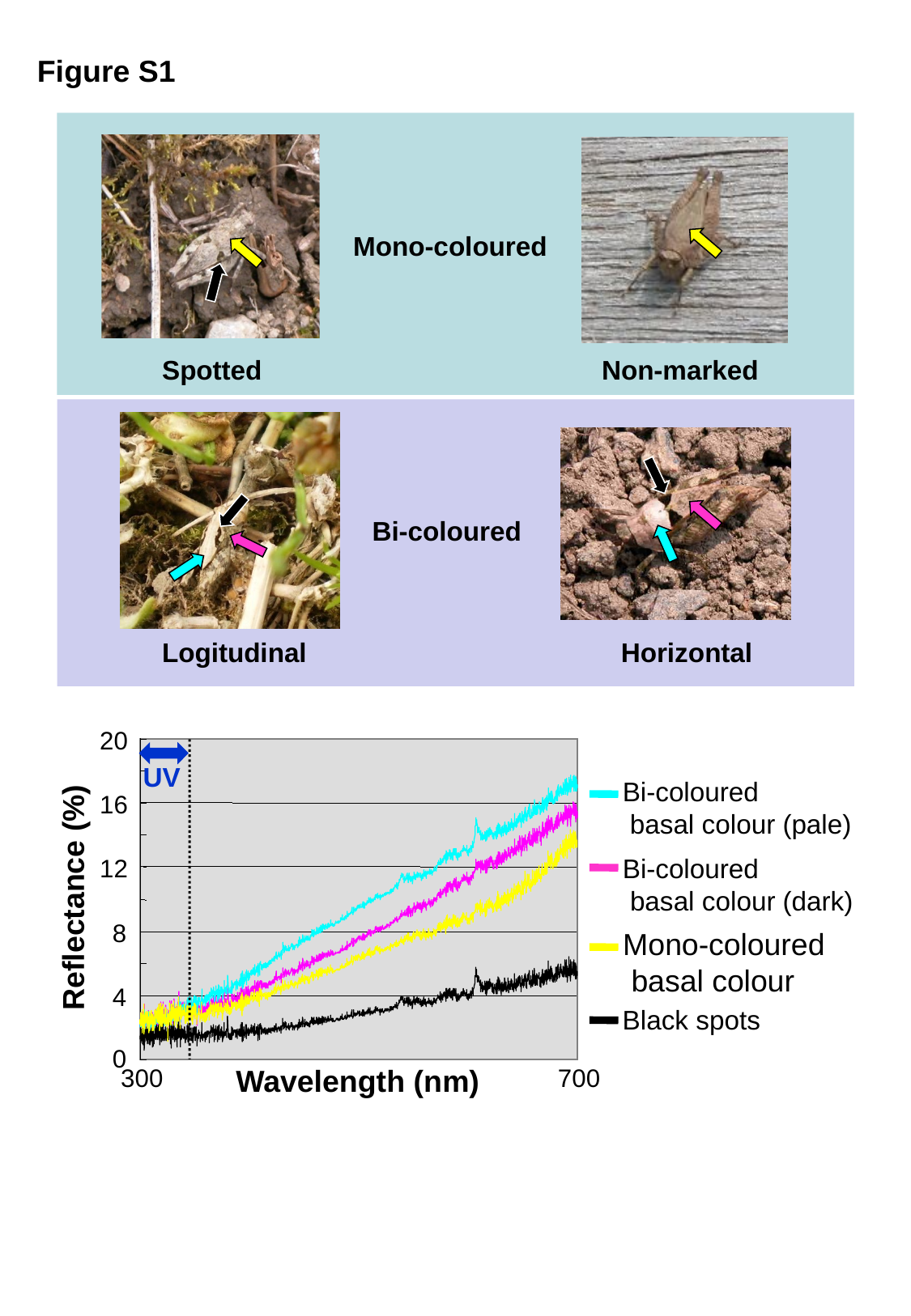

Figure S1
Mono-coloured
Spotted
Non-marked
Bi-coloured
Logitudinal
Horizontal
20
UV
Bi-coloured
 basal colour (pale)
16
Bi-coloured
 basal colour (dark)
12
Reflectance (%)
8
Mono-coloured
 basal colour
4
Black spots
0
Wavelength (nm)
700
300
